# The critical role of T cells in glucocorticoid-induced osteoporosis

**DOI:** 10.1038/s41419-020-03249-4

**Published:** 2020-12-14

**Authors:** Lin Song, Lijuan Cao, Rui Liu, Hui Ma, Yanan Li, Qianwen Shang, Zhiyuan Zheng, Liying Zhang, Wen Zhang, Yuyi Han, Xiaoren Zhang, Huilin Yang, Ying Wang, Gerry Melino, Changshun Shao, Yufang Shi

**Affiliations:** 1 The First Affiliated Hospital of Soochow University, State Key Laboratory of Radiation Medicine and Protection, Institutes for Translational Medicine, Soochow University Medical College, Suzhou, Jiangsu 215123 China; 2 CAS Key Laboratory of Tissue Microenvironment and Tumor, Shanghai Institute of Nutrition and Health, Shanghai Institutes for Biological Sciences, University of Chinese Academy of Sciences, Chinese Academy of Sciences, 200031 Shanghai, China; 3 Department of Experimental Medicine, TOR, University of Rome Tor Vergata, Rome, Italy

**Keywords:** Mechanisms of disease, Osteoimmunology

## Abstract

Glucocorticoids (GC) are widely used clinically, despite the presence of significant side effects, including glucocorticoid-induced osteoporosis (GIOP). While GC are believed to act directly on osteoblasts and osteoclasts to promote osteoporosis, the detailed underlying molecular mechanism of GC-induced osteoporosis is still not fully elucidated. Here, we show that lymphocytes play a pivotal role in regulating GC-induced osteoporosis. We show that GIOP could not be induced in SCID mice that lack T cells, but it could be re-established by adoptive transfer of splenic T cells from wild-type mice. As expected, T cells in the periphery are greatly reduced by GC; instead, they accumulate in the bone marrow where they are protected from GC-induced apoptosis. These bone marrow T cells in GC-treated mice express high steady-state levels of NF-κB receptor activator ligand (RANKL), which promotes the formation and maturation of osteoclasts and induces osteoporosis. Taken together, these findings reveal a critical role for T cells in GIOP.

## Introduction

Due to their powerful immunosuppressive ability, glucocorticoids (GC) are widely used in clinical practice to manage various autoimmune diseases and hyperinflammatory conditions. Prolonged administration of GC, however, has various adverse side effects, including loss of bone density; in fact, about 20% of all osteoporosis cases are a result of glucocorticoid administration^1^. Besides the physical debilitation and cost of medical care, osteoporosis is often associated with pain and increased risk of fractures^2^. Under normal physiological conditions, the process of bone remodeling involves both osteoclast-mediated bone resorption and osteoblast-mediated bone rebuilding. Accordingly, bone homeostasis is disrupted if there is an imbalance between bone resorption and bone reconstruction by these two cell types, resulting in abnormal bone remodeling. The differentiation process of osteoclasts from monocytes/macrophages, and their subsequent maturation, is well-studied and is known to be regulated by NF-κB receptor activator ligand (RANKL), its receptor RANK, and the decoy receptor osteoprotegerin (OPG)^3,4^.

Previous studies of glucocorticoid-induced osteoporosis (GIOP) have focused largely on the direct effects of GC on osteoblasts and their precursors, mesenchymal stem cells (MSCs), and osteoclasts. GC have been reported to induce apoptosis in osteoblasts and osteocytes^5^, and to directly impair the differentiation and function of osteoblasts^6^. There is increasing evidence that the immune system, through T and B lymphocytes, dendritic cells (DC), and cytokines, can modulate bone resorption and bone formation^7,8^. In some inflammatory bone diseases, inappropriately activated T cells can affect osteoclast maturation by production of IFN-γ, IL-4, and RANKL^9,10^. OPG, CD40 ligand (CD40L), and some cytokines, such as IL-6, TNF-α, IL-1, and IL-17, are also known to be involved in early bone rebuilding during fracture healing^11–13^. These studies strongly support a paradigm that the immune system, via the action of cytokines and immune cells, is actively involved in bone homeostasis.

Glucocorticoids are known to dampen a variety of immune cells, especially T and B lymphocytes^14,15^, an effect likely exerted through inhibition of proliferation and induction of apoptosis. Profound but transient peripheral lymphocytopenia usually occurs upon administration of GC^16^. Yet, the role of immune cells, especially T and B lymphocytes, in GIOP has not been specifically investigated. Since a decline in lymphocyte numbers usually accompanies the loss of bone density seen in GIOP, we hypothesized that lymphocytes also contribute to the maintenance of bone homeostasis. Therefore, we established a dexamethasone (Dex)-induced osteoporosis mouse model and examined the role of lymphocytes in osteoporosis. Interestingly, we found that T cells actually mediate the bone-depleting effects of Dex, and that RANKL expression by T cells is upregulated by Dex. Importantly, we also observed that while T cells in the periphery were greatly reduced in numbers by Dex, they were found to accumulate in the bone marrow, where they were protected from apoptosis. These findings demonstrate a novel pathogenic role for T cells in GIOP.

## Results

### Dexamethasone treatment depletes BMD

**Fig. 1:**
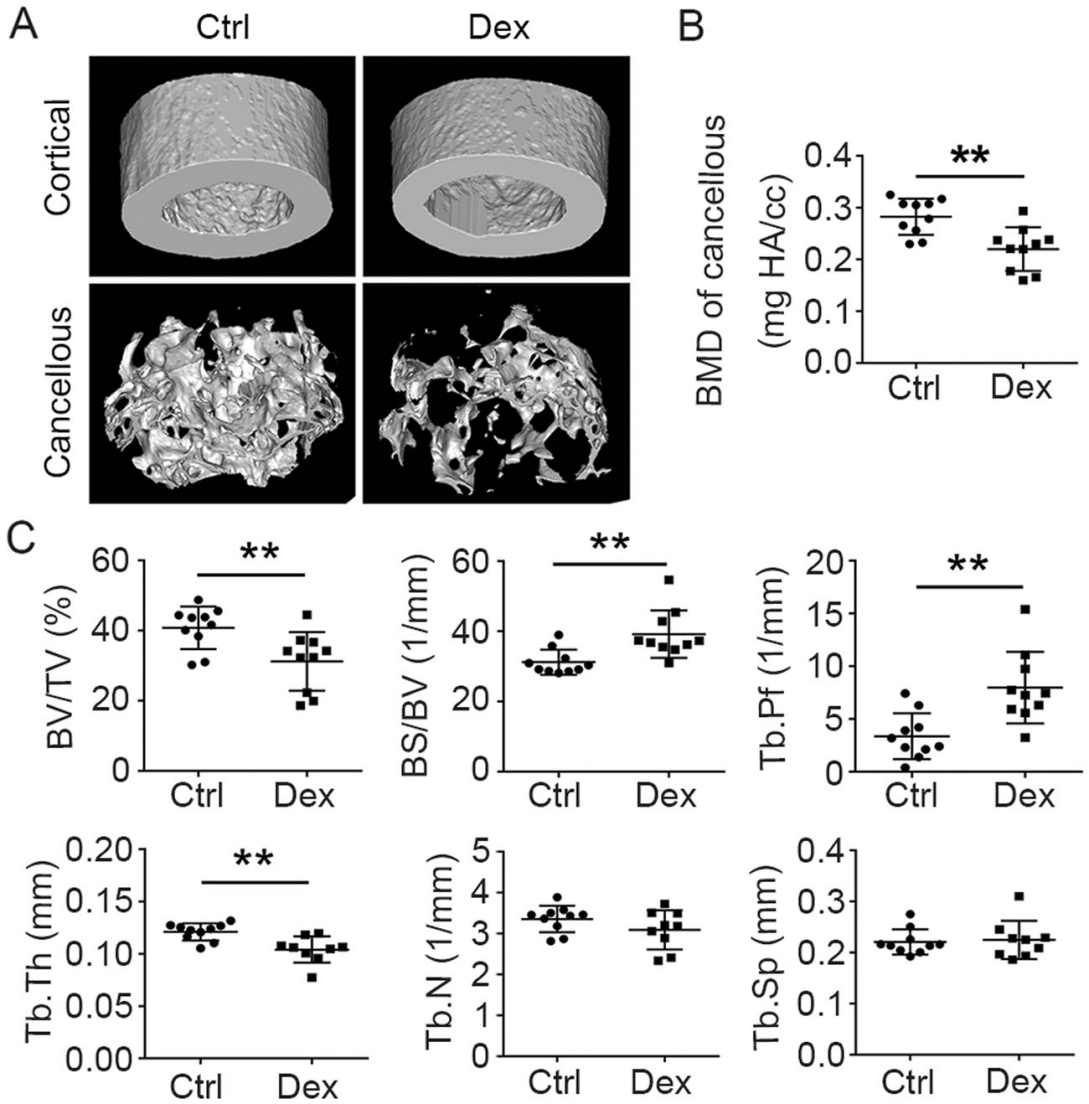
Bone mineral density decreases after dexamethasone treatment. Micro-CT analysis of femoral bones from WT mice (13–14-week-old females) treated with or without Dex (4 weeks of subcutaneous daily injection). **A** Images of the cortical (top) and cancellous bone (bottom). **B** Cancellous bone parameters were quantitated and compared between WT mice treated with Dex and untreated controls. BMD bone mineral density. **C** BV/TV bone-volume/tissue-volume ratio, BS/BV bone surface/volume ratio, Tb.Pf trabecular pattern factor, Tb.Th trabecular thickness, Tb.N trabecular number, Tb.Sp trabecular separation. Bars represent mean ± SD (*n* = 10). *p < 0.05; **p < 0.01; ***p < 0.001; ****p < 0.0001.

We first established a glucocorticoid-induced osteoporosis mouse model by injecting Dex (25 mg/kg body weight) daily for four weeks. Femurs from Dex-treated and control female mice were analyzed by microcomputed tomography (μCT). After 4 weeks of Dex administration, there was a significant loss of bone mass (Fig. 1A). BMD was also significantly decreased (Fig. 1B), as characterized by lower bone-volume/tissue-volume ratio (BV/TV) in trabecula, reduced trabecular thickness (Tb.Th), and higher bone surface/volume ratio (BS/BV) (Fig. 1C). Thus, Dex induced significant bone loss in our mouse model in vivo. As expected, we also observed a significant decrease in spleen size after 4 weeks of Dex injection (see SI Appendix, Fig. S1A), while the absolute numbers of B and T cells in spleen were also drastically reduced (see SI Appendix, Fig. S1B), findings that are indicative of lymphocytopenia.

### Mice devoid of T cells are protected from GIOP

**Fig. 2:**
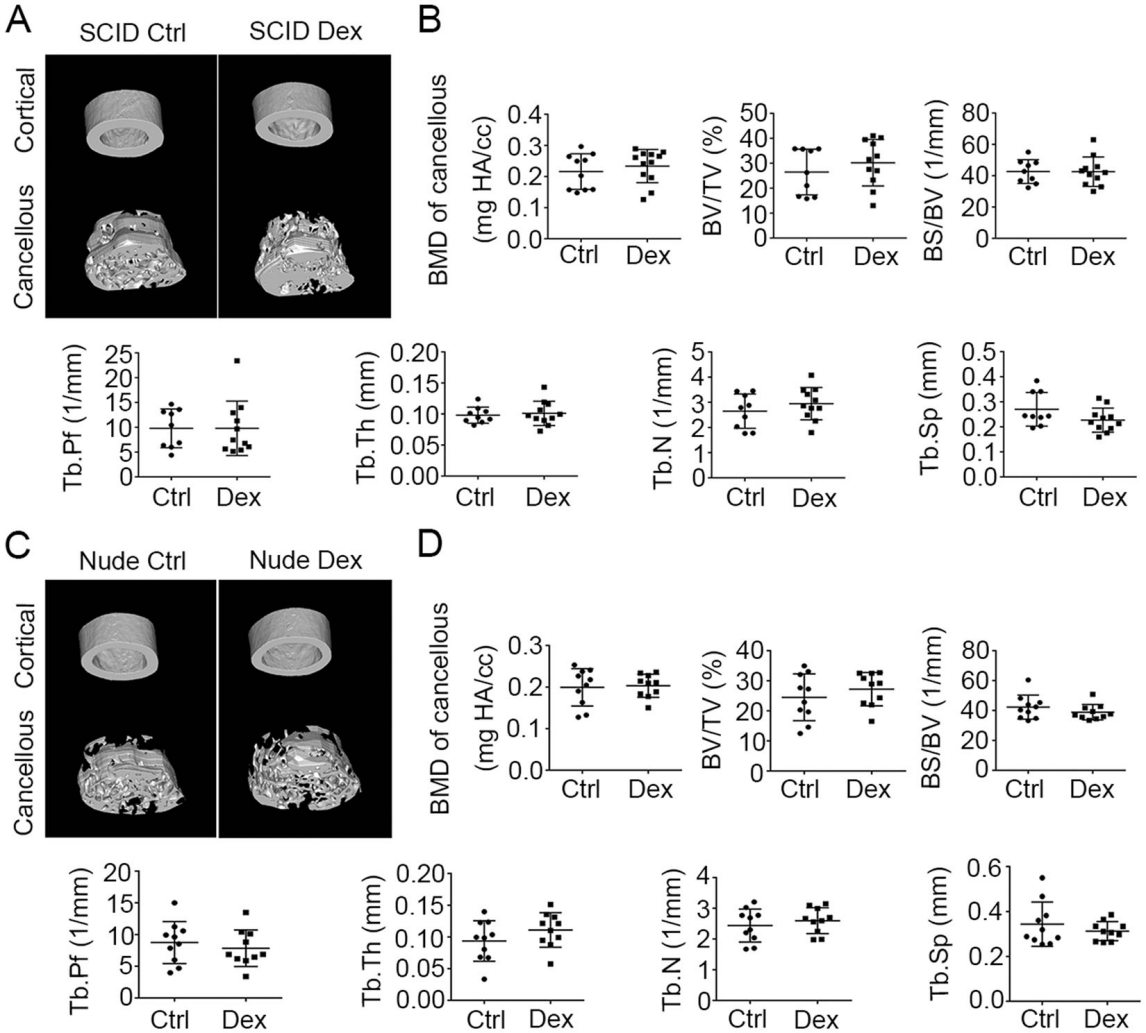
Mice lacking T cells are resistant to dexamethasone-induced osteoporosis. Micro-CT analysis of femoral bones from SCID and nude mice (13–14-week-old females) treated with or without Dex (4 weeks of subcutaneous injection). **A** Images of the cortical (top) and cancellous bone (bottom). **B** Cancellous bone parameters were quantitated and compared (as in Fig. 1) between SCID mice treated with Dex (*n* = 11) and untreated controls (*n* = 9). **C** Images of the cortical (top) and cancellous bone (bottom). **D** Cancellous bone parameters were quantitated and compared between nude mice treated with Dex (*n* = 11) and untreated controls (*n* = 10). Bars represent mean ± SD.

Since lymphocytes were greatly reduced in mice suffering from GIOP, we hypothesized that T and/or B lymphocytes may contribute directly to the maintenance of bone density. To investigate the role of lymphocytes in the development of GIOP, we utilized SCID mice that lack T and B cells (Fig. 2A). Contrary to our expectation, there were no significant differences in bone parameters between Dex-treated mice and controls, whether in femoral BMD, bone volume or surface, or indices of trabecular bone (Fig. 2B). This result strongly suggests that SCID mice are resistant to GIOP. We next evaluated the effect of Dex on BMD in nude mice, which are devoid of T cells, and found that these mice were also resistant to GIOP (Fig. 2C, D). These results suggest that GIOP is not a consequence of the decline of T and B lymphocytes, but conversely, the lymphocytes contribute to the development of GIOP.

### SCID mice reconstituted with T cells develop GIOP

**Fig. 3:**
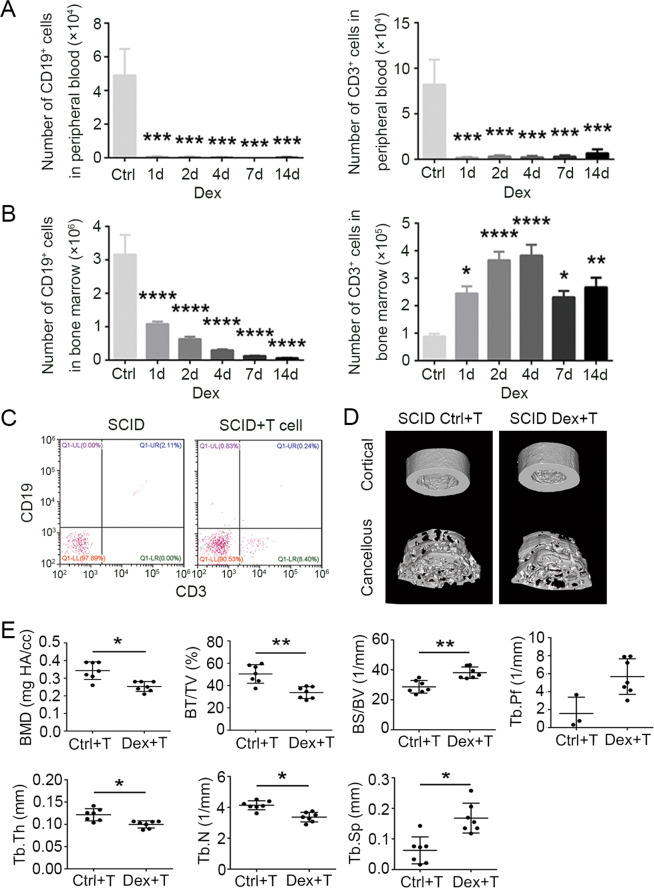
Reconstitution of SCID mice by transplantation of T cells re-establishes dexamethasone-induced osteoporosis. Numbers of CD19^+^ and CD3^+^ cells in peripheral blood (**A**) and bone marrow (**B**) obtained on days 1, 2, 4, 7, and 14 from WT mice treated with or without Dex were examined by immunofluorescence staining and flow cytometry (*n* = 5). **C** For reconstitution of SCID mice by T-cell transplantation, splenic T cells were isolated from BALB/c mice; then cells (5 × 10^6^) were transplanted into SCID mice by intravenous injection. CD3^+^ cells in bone marrow were fluorescence-stained and examined by flow cytometry after 4 weeks. **D** Images of the cortical (top) and cancellous bone (bottom). **E** Cancellous bone parameters were quantitated and compared (as in Fig. 1) between T-cell-transplanted SCID mice treated with or without Dex (*n* = 7) for 4 weeks. Bars represent mean ± SD.

GC are known to be potent inducers of apoptosis in peripheral T cells. Indeed, flow-cytometric analysis showed that the numbers of lymphocytes in peripheral blood of WT mice decreased remarkably within 1 day of starting Dex treatment (Fig. 3A). Surprisingly, in bone marrow, the number of T cells actually *increased* several-fold following Dex administration, and remained high for 2 weeks (Fig. 3B). In contrast, B-cell numbers in bone marrow were found to decrease after Dex treatment (Fig. 3B), like those in peripheral blood. These results suggest that T cells in the bone marrow may actually be involved in GIOP induction. To determine whether T cells can indeed mediate GIOP, we reconstituted SCID mice with T cells and examined the effect on GIOP induction. Splenic T cells isolated from wild-type (WT) mice were transplanted into SCID mice via tail-vein injection in two doses of 5 × 10^6^ cells 7 days apart, resulting in detectable numbers of bone marrow T cells (Fig. 3C). When these mice were subjected to Dex treatment, typical signs of bone loss developed, demonstrating that SCID mice reconstituted with T cells are susceptible to GIOP (Fig. 3D, E). These data suggest that T cells that accumulate in the bone marrow in Dex-treated mice may play a key role in the development of GIOP.

### RANKL produced by T cells mediates GIOP

**Fig. 4:**
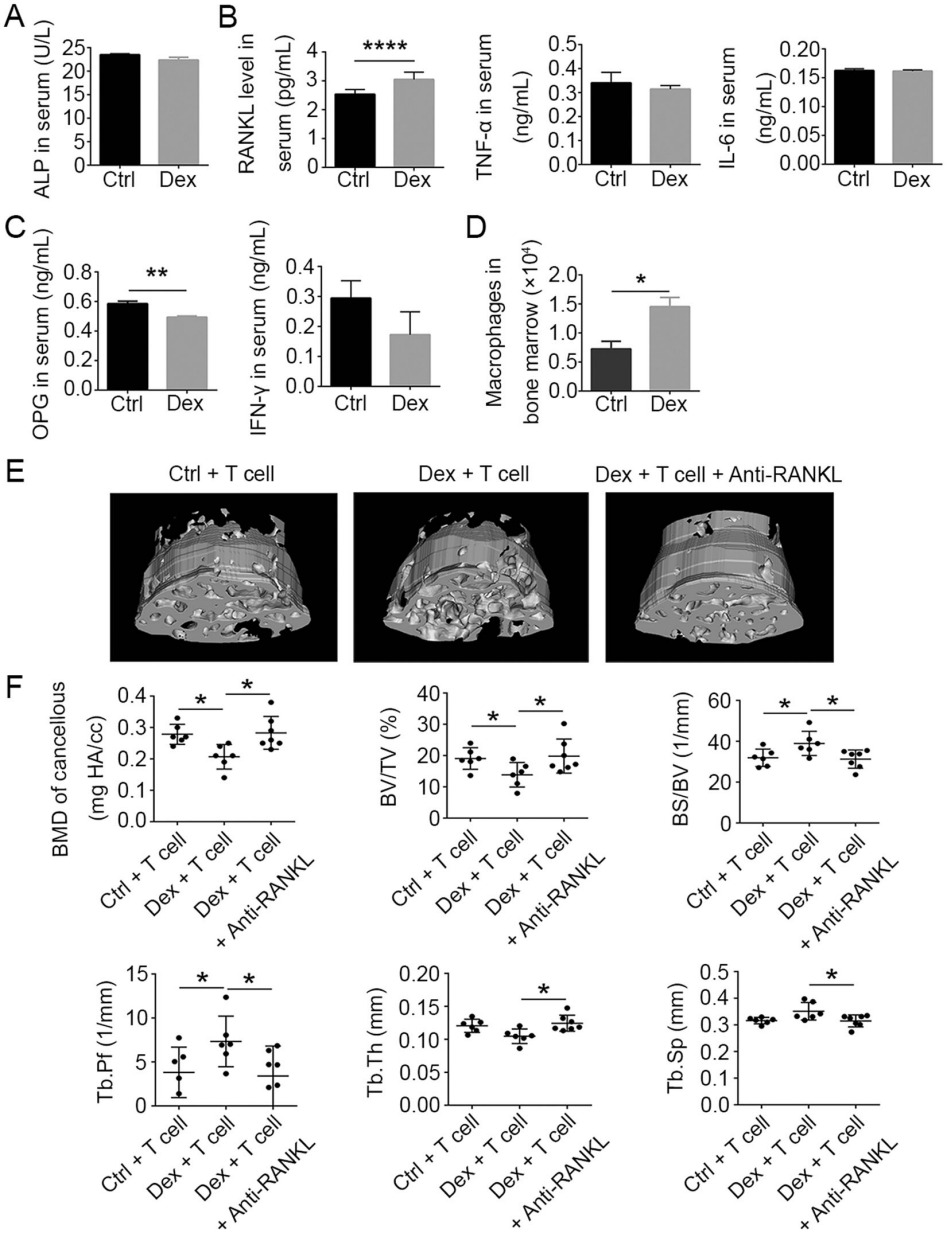
Upregulation of RANKL in T cells mediates dexamethasone-induced osteoporosis. **A** Activity of alkaline phosphatase in serum from WT mice treated with or without Dex (*n* = 5). Protein levels of RANKL, TNF-α, and IL-6 (**B**) and OPG and IFN-γ (**C**) in serum from WT mice treated with or without Dex (*n* = 5). **D** Numbers of macrophages in bone marrow from WT mice treated with or without Dex on day 4 as analyzed by flow cytometry (*n* = 5). **E** Micro-CT images of the femoral bone cancellous bone from SCID + T cells, SCID + T + Dex, and SCID + T + Dex+aRANKL. **F** Cancellous bone parameters were quantitated and compared between T-cell-reconstituted SCID mice (*n* = 6), Dex-treated T-cell-reconstituted SCID mice (*n* = 6), and anti-RANKL-treated Dex-treated T-cell-reconstituted SCID mice (*n* = 7) after 4 weeks of treatment. Bars represent mean ± SD.

Considering the critical role of osteoblasts in osteogenesis, we examined the effects of Dex administration on osteoblast numbers in the bone marrow using H&E staining, and on the levels of alkaline phosphatase (ALP), a key functional marker of osteoblast activity, using enzyme-linked immunosorbent assay (ELISA) of peripheral blood in WT mice. Neither osteoblast numbers nor ALP protein levels were affected by Dex treatment compared to untreated controls (Fig. 4A, see SI Appendix, Fig. S2A).

The effects of Dex on several osteoclast-modulating cytokines were then tested. RANKL acts as a critical regulator of osteoclast differentiation and maturation from monocytes/macrophages. The steady-state protein levels of RANKL in the serum, as measured by ELISA, significantly increased after 4 weeks of continuous Dex treatment (Fig. 4B). TNF-α and IL-6, which have been previously reported to promote bone resorption^13^, were found to be unaffected at the serum protein level upon Dex treatment (Fig. 4B).

OPG, as a decoy receptor of RANKL, hinders the binding of RANKL to RANK, thus negatively regulating the RANKL–RANK signaling pathway. IFN-γ can induce rapid protein degradation of the RANK signaling adapter protein, tumor necrosis factor receptor-associated factor 6 (TRAF6), and thus inhibit RANKL-induced activation of osteoclasts^17^. When we measured the protein levels of OPG and IFN-γ in the serum of Dex-treated mice, OPG was found to be decreased, while IFN-γ was not significantly affected (Fig. 4C). The Dex-induced decrease in OPG levels could presumably augment the RANKL–RANK signaling pathway and thereby promote the differentiation and maturation of osteoclasts. Interestingly, the total number of CD11b^+^ F4/80^+^ macrophages, from which osteoclasts are derived, was significantly increased in bone marrow after Dex treatment (Fig. 4D). In addition, Dex treatment resulted in greater numbers of tartrate-resistant acid phosphatase (TRAP)-positive cells in the femoral bone marrow (see SI Appendix, Fig. S2B). Accordingly, elevated numbers of TRAP-positive cells were also observed in bone marrow cultured from Dex-treated mice, when compared to untreated controls (see SI Appendix, Fig. S2C). To confirm that T cells are the source of the upregulated RANKL levels that mediate GIOP, we transplanted WT splenic T cells into SCID mice, and attempted to induce GIOP using Dex treatment. A third group was also administered anti-RANKL antibody every other day during the duration of Dex treatment in order to block signaling via the RANKL–RANK pathway. The effects on several bone parameters as determined by μCT analysis of trabecular bone revealed that anti-RANKL treatment completely reversed the Dex-induced bone loss (Fig. 4E, F). These results show that the GIOP in SCID mice reconstituted with donor T cells is completely abrogated by antibody blockade of RANK signaling. Together, these findings strongly suggest that GIOP is mediated by RANKL expressed by T cells.

### Dexamethasone-induced RANKL expression occurs preferentially in T cells

**Fig. 5:**
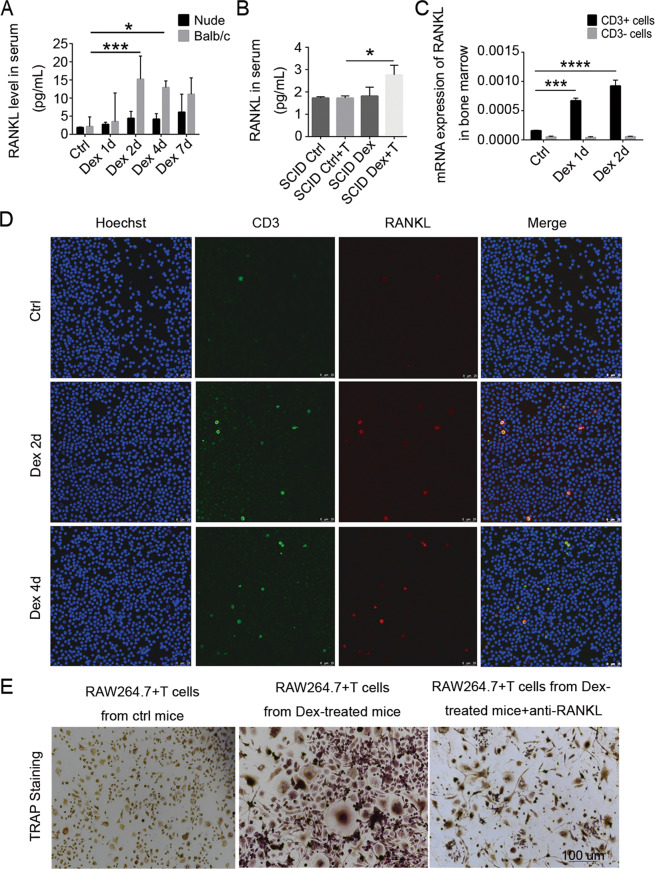
Dexamethasone preferentially stimulates RANKL expression in T cells. **A** RANKL protein levels in serum from WT and nude mice treated with or without Dex, after 1, 2, 4, or 7 days (*n* = 4). **B** RANKL protein levels in serum from SCID mice with or without T-cell reconstitution, treated with or without Dex for 4 weeks (*n* = 5). Bars represent mean ± SD. **C** CD3^+^ and CD3^−^ cells were isolated from bone marrow 1 or 2 days after Dex injection, and RANKL mRNA levels (relative to beta-actin) were analyzed by Q-PCR. **D** Immunofluorescence staining of CD3 and RANKL in bone marrow cells from WT mice treated with or without Dex. Scale bar, 25 μm. **E** Bone marrow T cells from mice treated with or without Dex were isolated, and cocultured with the mouse monocyte/macrophage cell line RAW264.7 and M-CSF for 10 days. The RAW264.7 cells were then stained for tartrate-resistant acid phosphatase to identify osteoclasts and a nuclear counterstain. Scale bar, 100 μm.

To determine whether GC upregulate RANKL specifically in T cells, we measured the serum levels of RANKL after Dex treatment of mice with different constitutions of T and B cells. While Dex treatment upregulated RANKL expression in WT Balb/c mice (having both T and B cells), no increase was observed in nude mice (lacking T cells) (Fig. 5A) or SCID mice (deficient in both T and B cells) (Fig. 5B). However, after adoptive transfer of T cells from WT mice into SCID mice, RANKL expression was found to increase in response to Dex treatment (Fig. 5B). When CD3^+^ or CD3^−^ cells, respectively, were isolated from bone marrow and analyzed for RANKL transcripts, only the CD3^+^ cells were found to upregulate RANKL in response to Dex treatment (Fig. 5C). Confocal laser scanning analysis of bone marrow smears revealed that Dex treatment resulted in more RANKL being co-localized with CD3 on T cells (Fig. 5D).

We next tested whether Dex-treated T cells can promote the differentiation of osteoclasts in vitro. T cells isolated from bone marrow of control or Dex-treated mice were cocultured with RAW264.7 cells, a monocyte/macrophage line capable of differentiating into osteoclasts. After 10 days, the matured osteoclasts were stained for TRAP. T cells from Dex-treated mice had a much greater capacity to induce osteoclast differentiation than did those from control mice (Fig. 5E). This Dex-enhanced osteoclast growth, however, was effectively blocked by neutralizing antibody against RANKL (Fig. 5E, see SI Appendix, Fig. S3A, B).

We further verified that RANKL could be upregulated by Dex using isolated splenic T cells and T hybridoma A1.1 cells in vitro (see SI Appendix, Fig. S3C–E). We found that Dex-induced enhancement of RANKL expression was completely reverted by glucocorticoid receptor (GR) inhibitor RU486 (see SI Appendix, Fig. S3F). Together, these results clearly demonstrate that Dex-induced upregulation of RANKL expression occurs preferentially in T cells.

Increased extracellular calcium levels have been proposed to modulate bone remodeling. The calcineurin/NFAT signaling pathway reportedly plays a role in regulating RANKL expression^18^. We found that RANKL expression was significantly suppressed by calcineurin inhibitors (see SI Appendix, Fig. S3G), further proving that this signaling pathway is involved in the regulation of RANKL expression.

### T cells in bone marrow are protected from GC-induced apoptosis

**Fig. 6:**
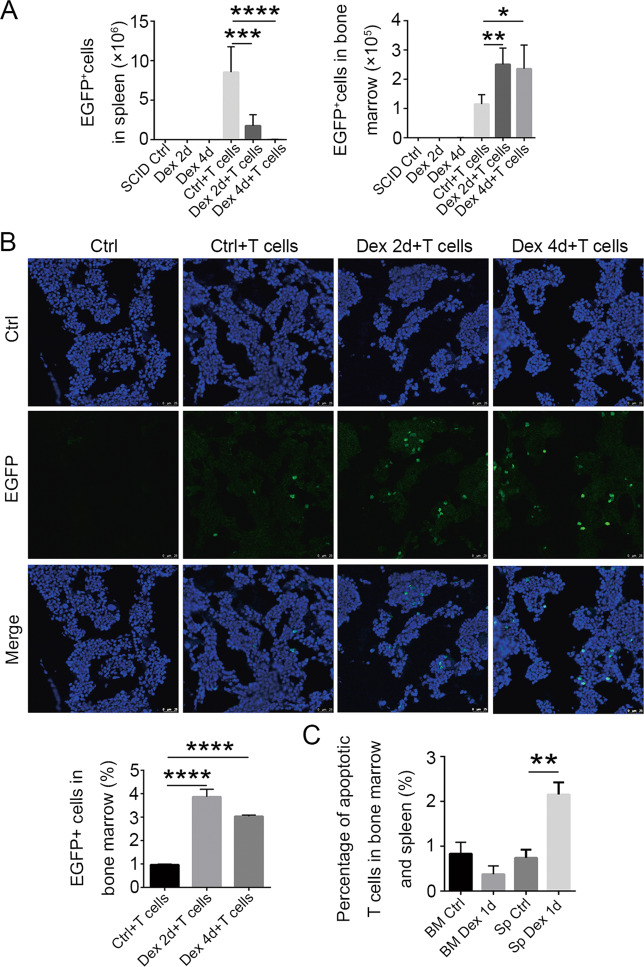
T cells accumulate in bone marrow after GC treatment. **A** Splenic T cells were isolated from 10-week-old EGFP mice, and transferred into SCID mice. The splenic and bone marrow EGFP^+^ cells from SCID mice were quantitated by flow cytometry at 2 and 4 days after Dex injection (*n* = 3). **B** Fluorescence microscopy showing T-cell accumulation in bone marrow. Scale bar, 25 μm. Statistical histograms are shown below, left. **C** Percentages of apoptotic T cells in spleen and bone marrow, as detected by flow cytometry after staining with 7-AAD and Annexin V (*n* = 5). Bars represent mean ± SD.

We speculated that several mechanisms may be responsible for the enhanced accumulation of T cells in the bone marrow following Dex treatment: increased homing to and sequestration in the bone marrow, increased proliferation, or decreased apoptosis in the bone marrow. We therefore tested these possibilities by isolating splenic CD3^+^ cells from EGFP mice, and transplanting them into SCID mice by intravenous injection. The mice were then treated with Dex, and cells from the spleen and bone marrow were analyzed by flow cytometry. We found that while EGFP^+^ T cells in the periphery were reduced in numbers by Dex treatment, they actually appeared at higher numbers in the bone marrow (Fig. 6A). Immunofluorescence staining of bone marrow sections showed similar results (Fig. 6B). To investigate whether Dex treatment could induce the proliferation of T cells in the bone marrow, we stained proliferating T cells with Ki67 antibody. No Ki67^+^ cells could be observed after Dex treatment, indicating that little proliferation occurred (see SI Appendix, Fig. S4A). Furthermore, the cell cycle profile as revealed by DNA content, and the percentage of T cells in the summed cycling phases (S + G2/M) of the cell cycle, were both unaffected by Dex treatment (see SI Appendix, Fig. S4B), again ruling out enhanced proliferation. These results indicate that the increased accumulation of T cells in bone marrow resulting from Dex treatment is not due to increased local proliferation.

To determine if bone marrow T cells are protected from GC-induced apoptosis, we looked for differential Dex-induced T-cell apoptosis in the bone marrow and periphery. The percentage of apoptotic T cells was found to be significantly increased in the spleen after Dex treatment, but it remained unchanged or possibly reduced in the bone marrow (Fig. 6C, see SI Appendix, Fig. S4C), indicating that the bone marrow tissue microenvironment can protect T cells from Dex-induced apoptosis. Recently, Dex has been shown to increase the expression of the anti-apoptotic protein BCL-2 in bone marrow T cells^19^. We therefore examined the expression of several apoptosis-related genes in T cells from Dex-treated mice, and found that both pro-apoptotic and anti-apoptotic genes were upregulated (see SI Appendix, Fig. S4D). Thus, it remains to be determined how T cells in bone marrow become resistant to apoptosis.

### Chemokines drive the accumulation of T cells in bone marrow

**Fig. 7:**
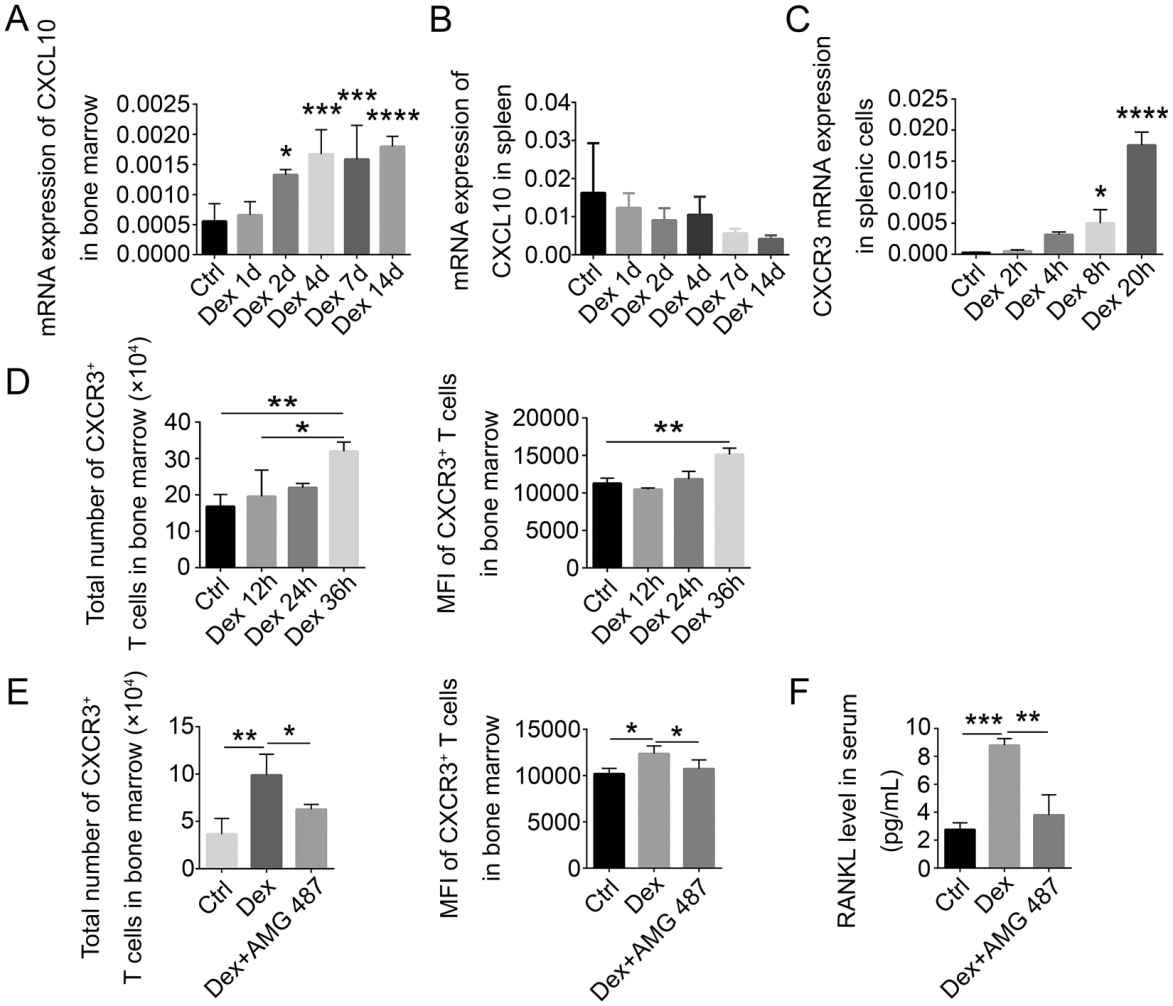
Chemokines drive the accumulation of T cells in bone marrow. CXCL10 mRNA levels relative to beta-actin in bone marrow (**A**) and spleen (**B**) from WT mice treated with Dex for the indicated times, as measured by Q-PCR (*n* = 5). **C** CXCR3 mRNA levels (relative to beta-actin) in splenic T cells from WT mice treated with Dex for the indicated times (*n* = 5). **D** The total number of CXCR3-expressing cells and median fluorescence intensity of CXCR3 in bone marrow T cells from WT mice treated with Dex for the indicated times, as measured by flow cytometry (*n* = 5). **E** The total number of CXCR3-expressing cells and median fluorescence intensity of CXCR3 in bone marrow T cells from WT mice treated with or without Dex, with or without injection of CXCR3 antagonist (5 mg/kg daily, for 3 days) (*n* = 5). **F** RANKL protein levels in serum from WT mice treated as in (**E**) (*n* = 4). Bars represent mean ± SD.

The above results show that T cells preferentially accumulate in the bone marrow after Dex treatment. Since this mobilization of T cells to the bone marrow might be driven by chemokines, we tested whether increased chemokine signals from the bone marrow are responsible for T-cell accumulation there. We found that CXCL10 expression levels in bone marrow rose during Dex treatment (Fig. 7A), while its expression in the spleen gradually declined (Fig. 7B). Interestingly, T-cell expression of CXCR3 transcripts (a CXCL10 receptor) was also upregulated by Dex (Fig. 7C). Both the absolute numbers of CXCR3^+^ T cells in bone marrow and the amount of CXCR3 per cell were also enhanced by Dex treatment (Fig. 7D). Furthermore, the Dex-induced increase in accumulated CXCR3^+^ T cells was attenuated by the CXCR3-specific antagonist AMG-487 (Fig. 7E). At the same time, the induction of RANKL by Dex was abrogated by injection of AMG-487 into the mice (Fig. 7F). These data suggest that the CXCL10–CXCR3 axis is likely to be involved in the migration of T cells from periphery to bone marrow.

## Discussion

GIOP is a major side effect of glucocorticoid application in clinical practice. Although many investigations have focused on this side effect, the underlying molecular mechanism is still not fully understood. Here, we demonstrated that Dex treatment preferentially induces T-cell expression of RANKL, an important induction factor for osteoclasts, and that mice lacking T cells are resistant to GIOP. While artificial introduction of T cells into SCID mice can re-establish GIOP, this effect is completely abrogated by the neutralization of RANKL, suggesting that T cells contribute to bone loss by producing RANKL. Interestingly, while the T cells are greatly reduced by Dex in the periphery, they increasingly accumulate in the bone marrow, where they are protected from Dex-induced apoptosis. The level of CXCL10, a specific chemotactic factor for T cells, was increased in bone marrow, while CXCR3, the receptor for CXCL10, was upregulated on T cells by Dex treatment, indicating that the accumulation of T cells in bone marrow is partially mediated by the CXCL10–CXCR3 axis. Thus, the increased accumulation in the bone marrow of T cells with greatly enhanced RANKL productivity results in increased development of osteoclasts from monocytes/macrophages, ultimately leading to bone loss and osteoporosis.

The nascent field of osteoimmunology explores the interactions between the skeletal and immune systems^20^. The crosstalk between these two systems may be best illustrated by the pathogenesis of rheumatoid arthritis, in which Th17 cells were found to induce the progressive destruction of multiple joints through aberrant expression of RANKL. However, there were reports showing that T cells could both positively and negatively regulate osteoclastogenesis under different physiological or pathological states by their secretion of RANKL, IL-17, OPG, IFN-γ, IL-4, and other factors^9,11,21,22^. At the same time, the interactions between T cells, osteoblasts, and osteoclasts can also regulate the secretion of osteoclast-promoting or osteoclast-inhibiting factors under certain conditions^12,23^. However, the role of T cells in GIOP has not been fully elucidated, probably due to the loss of lymphocytes observed in the spleen, lymph nodes, thymus, and other tissues after glucocorticoid treatment.

Bone marrow T cells are generally immature T cells or memory T cells preserved after an immune response. Mature T cells enter and exit bone marrow through blood vessels. We found that spleen-derived donor T cells could accumulate in bone marrow, and this increase of T-cell numbers in bone marrow is not due to an enhanced local proliferation, but rather to an increase in T-cell migration to bone marrow with Dex treatment. Interestingly, while Dex remarkably increased the percentage of apoptotic T cells in the periphery, T cells in bone marrow were protected from apoptosis. In the context of dietary restriction (DR), memory T-cell numbers collapsed in the secondary lymphoid organs, but remarkably accumulated in large numbers within the bone marrow, which provides a safe haven for T cells where they are protected from infections and tumors during energy challenges^19^. Interestingly, the redistribution of memory T cells from the periphery to the bone marrow during DR also depends on GC. It should be noted that while the CXCL12–CXCR4 axis was shown to contribute to T-cell homing during DR, our findings indicate that the CXCL10–CXCR3 axis was responsible for a large proportion of T-cell chemotaxis to the bone marrow during Dex treatment, since CXCR3 antagonist could significantly reduce the accumulation of these T cells in bone marrow. We may postulate that inhibiting this chemotactic signaling pathway could reduce bone loss, joint damage, and possibly even bone metastasis of tumor cells associated with the use of Dex. Further studies are needed to understand the various physiological and clinical implications of GC-driven increased T-cell accumulation in bone marrow. There are many questions, still, that need to be addressed: how does GC upregulate RANKL in T cells? How are T cells protected by the bone marrow microenvironment? How does the bone destruction in rheumatoid arthritis differ from GIOP? Answers to these questions will certainly further advance the field of osteoimmunology and provide new strategies for the prevention and treatment of osteoporosis.

## Materials and methods

### Animals and the GIOP model

Female Balb/c, nude, and SCID mice at 9–10 weeks of age were purchased from the Vital River Laboratory (Beijing, China). The mice were acclimated in the animal facility for at least 1 week before experimental procedures. All mouse experiments were in compliance with the institutional protocols on animal welfare and were approved by the Ethics Committee of Soochow University. For the GIOP model, phosphate-buffered saline (PBS) or Dex (Lianshui, Jiangsu, China) was injected subcutaneously at 25 mg/kg body weight daily for 4 weeks.

### Antibody blockade of RANKL in vivo

Monoclonal antibody specific for mouse RANKL (100 μg, clone IK22/5, BioXCell, West Lebanon, NH) was injected intraperitoneally every other day during the duration of Dex administration.

### Differentiation of mouse osteoclasts

Balb/c mice were treated with or without Dex for 2 or 7 days. RAW264.7 cells were cocultured with T cells in the presence of 33 ng/mL M-CSF for 10 days, and were then fixed and stained for tartrate-resistant acidic phosphatase (TRAP) using an acid phosphatase assay kit (Sigma-Aldrich, St. Louis, MO). TRAP-positive multinucleated (more than three nuclei) cells were scored as osteoclasts under a microscope at 20× magnification.

### Structural analysis of trabecular bone by micro-CT

After osteoporosis induction in the GIOP model, mice were euthanized and the femur and tibia of the right leg were taken, and joint integrity at both ends was carefully preserved. All soft tissues attached to the bone surface and joints were carefully removed, and then the bones were fixed in 4% paraformaldehyde and stored for future analysis. Three-dimensional images at a normal resolution of 9 μm were taken, using the Small Animal In Vivo Micro-Compute Tomography (SkyScan, Belgium) at the Orthopaedic Research Institute of Soochow University. Femoral bones were placed in a measuring vessel with the bone long axis perpendicular to the scanning plane, and scanning regions were taken from the distal growth plate. The X-ray source was set at 50 kV and 500 μA and the exposure time was 100 ms. The volume of interest (VOI) of the trabecular bone of the femur included 150 cross-sectional slices of the distal femur, beginning 60 slices up from the growth plate region. The VOI of the cortical bone included 100 cross-sectional slices of the distal femur beginning 350 slices up from the growth plate region. Parameters of bone mineral density (BMD) and bone-volume/tissue-volume ratio (BV/TV) were calculated using the Skyscan CT-analyzer software.

### T-cell transplantation

Donor Balb/c mice were euthanized and rinsed with 70% ethanol. Spleens were harvested and splenic T cells were isolated using the MojoSort^™^ kit (BioLegend, San Diego, CA). Cells (5 × 10^6^ cells in 200 μL of PBS per mouse) were injected intravenously into 9-week-old SCID mice.

### Enzyme-linked immunosorbent assay

Blood samples were collected from mice, and centrifuged at 3000×*g* for 20 min to separate the serum. Concentrations of RANKL and other cytokines in the serum were measured using ELISA kits (DLDEVELOP, Wuxi, China) according to the manufacturer’s protocol.

### Quantitative RT-PCR

Quantitative RT-PCR was performed to measure mRNA levels. Total RNA was extracted from A1.1 cells, purified T cells, or whole-nucleated bone marrow cells with Trizol Reagent (Sigma-Aldrich). First-strand cDNA was synthesized from total RNA and subsequently subjected to Real-Time PCR, which was performed on an Applied Biosystems^™^ QuantStudio^™^ 6 Flex Real-Time PCR System, using SYBR Select Master Mix (Thermo Fisher, Waltham, MA). The primer sequences were as follows:

RANKL-forward (F) 5′-CAACATATCGTTGGATCACAGCA-3′,

RANKL-reverse (R) 5′-GACAGACTCACTTTATGGGAACC-3′;

CXCL10-F 5′-CCAAGTGCTGCCGTCATTTTC-3′,

CXCL10-R 5′-GGCTCGCAGGGATGATTTCA-3′;

BIM-F 5′-CCCGGAGATACGGATTGCAC-3′,

BIM-R 5′-GCCTCGCGGTAATCATTTGC-3′;

BAX-F 5′-TGAAGACAGGGGCCTTTTTG-3′,

BAX-R 5′-AATTCGCCGGAGACACTCG-3′;

BAK-F 5′-GTGACCTGCTTTTTGGCTGAT-3′,

BAK-R 5′-GGTCTCTACGCAAATTCAGGG-3′;

BAD-F 5′-AAGTCCGATCCCGGAATCC-3′,

BAD-R 5′-GCTCACTCGGCTCAAACTCT-3′;

BCL-2-F 5′-ATGCCTTTGTGGAACTATATGGC-3′,

BCL-2-R 5′-GTATGCACCCAGAGTGATGC-3′;

BCL-XL-F 5′-GACAAGGAGATGCAGGTATTGG-3′,

BCL-XL-R 5′-CCCGTAGAGATCCACAAAAGT-3′;

MCL1-F 5′-CAAAGATGGCGTAACAAACTGG-3′,

MCL1-R 5′-CCGTTTCGTCCTTACAAGAACA-3′;

Actin-F 5′-CAACGAGCGGTTCCGATG-3′,

Actin-R 5′-GCCACAGGATTCCATACCCA-3′.

Actin was used as the reference to normalize each sample for quantification.

### Cell cycle analysis by flow cytometry

Cells were harvested and washed once with PBS, fixed in 70% ethanol, and resuspended in staining buffer consisting of 50 µg/ml propidium iodide and 10 µg/ml RNase A in PBS. Following a 30-min incubation at 37 °C in the dark, samples were analyzed by flow cytometry (CytoFLEX, Beckman-Coulter).

### Cell isolation

Bone marrow cells were harvested from the femur and tibia by flushing the bone marrow cavity with 1 mL of PBS. Spleen and blood were also collected. Erythrocytes were depleted by hemolysis (Tris-NH_4_Cl) and debris was removed by passing through a 70-μm mesh strainer.

### Analysis of cell surface markers by flow cytometry

Cells were stained with fluorescently conjugated antibodies for 30 min, washed twice, and then resuspended in 200 μL of PBS, all at 4 °C. The antibodies were as follows: Pacific Blue^™^ anti-CD3 (Biolegend), PE/Cy7 anti-CD183 (Biolegend), PerCP/Cy5.5 anti-CD195 (Biolegend), APC anti-CD54 (Biolegend), APC anti-F4/80 (eBioscience, San Diego, CA), and Brilliant Violet 605^™^ anti-CD19 (Biolegend).

### Immunofluorescence and histochemical staining

Mouse femoral bones were fixed in 4% PFA for 2 h at 4 °C, decalcified with 0.5 M EDTA (Sigma-Aldrich) for 4 weeks, and decalcified bones were immersed in a 20% sucrose and 2% polyvinylpyrrolidone (Sigma-Aldrich) solution for 1 week.

For immunostaining, the bones were embedded and frozen in OCT, and 6-μm-thick sections were cut along the longitudinal axis of the femur. Bone sections were blocked in 5% bovine serum albumin and 0.3% Triton X-100 for 1 h, and incubated with antibodies (1:300 for primary antibodies, 1:1000 for secondary antibodies). Nuclei were counterstained with DAPI. The antibodies were as follows: anti-CD3 (abcam, Cambridge, MA), anti-RANKL (abcam), Alexa Fluor 555 Donkey Anti-Rabbit IgG H&L, and Alexa Fluor 488 Goat Anti-Rat IgG H&L (abcam).

To detect osteoblasts and osteoclasts, the tissues were dehydrated in 75% ethanol overnight, then sequentially in 85% ethanol, 95% ethanol, and 100% ethanol for 1 h each. The femoral bones were treated with xylene twice for 15 min, then embedded in paraffin, and 5-μm-thick sections were taken. Morphological analysis of osteoblasts was performed by standard hematoxylin and eosin (H&E) staining. TRAP was stained using an acid phosphatase assay kit (Sigma-Aldrich), and TRAP-positive osteoclasts were examined at 20× magnification.

### Statistical analysis

Datasets were compared using two-tailed Student’s *t* test or one-way ANOVA. Data were analyzed using Graph Pad Prism software version 6.0. All values are reported as mean ± SD, with significance indicated as *p < 0.05, **p < 0.01, ***p < 0.001, ****p < 0.0001.

## Supplementary information

Supplementary Figure Legends

FIG 1s

FIG 2s

FIG 3s

FIG 4s
