# Supplementary material for: The critical role of T cells in glucocorticoid-induced osteoporosis
Source: Cell Death Dis. 2020 Dec 14;12(1):45. doi: 10.1038/s41419-020-03249-4 (PMC7791068; doi:10.1038/s41419-020-03249-4)
Supplement: Supplementary file 1 — Supplementary Figure Legends [file 41419_2020_3249_MOESM1_ESM.docx]

**Supplementary Information for**

**The Critical Role of T Cells in Glucocorticoid-induced Osteoporosis**

Lin Song^1#^, Lijuan Cao^1#^, Rui Liu^1^, Hui Ma^1^, Yanan Li^1^, Qianwen Shang^1^, Zhiyuan Zheng^1^, Liying Zhang^1^, Yuyi Han^1^, Wen Zhang^1^, Xiaoren Zhang^2^, Huilin Yang^1^, Ying Wang^2^, Gerry Melino^3^, Changshun Shao^1^*, and Yufang Shi^1,2,3^*.

^*^Corresponding authors: Changshun Shao or Yufang Shi

Email: shaoc@suda.edu.cn or yfshi@suda.edu.cn

**This file includes:** Legends for Datasets S1 to S4

**FIGURE S1. Dexamethasone causes lymphocytopenia**

(A) Gross images of spleens in control and Dex-treated mice

(B) Splenic CD19^+^ and CD3^+^ cell counts in control and Dex-treated mice, n = 5, *p < 0.05

**FIGURE S2. Dexamethasone increases the abundance of osteoclasts (TRAP-positive cells)**

(A) Femora of Dex-injected (25 mg/kg body weight, daily for 4 weeks) and control mice were collected, HE staining of decalcified femora sections were performed for examination of osteoblasts in Dex-injected and control mice.

(B) Femora of Dex-injected (4 weeks) and control mice were collected, and stained for tartrate-resistant acid phosphatase that marks osteoclasts, combined with nuclear counter staining. Scale bars, 250 μm.

(C) Bone marrow from ctrl and Dex-injected mice were isolated, cultured for 10 days, then stained with tartrate-resistant acid phosphatase for osteoclasts, combined with nuclear counter staining. Scale bars, 100 μm. ***p < 0.001

**FIGURE S3. Dexamethasone induces RANKL expression in T cells**

(A) RANKL protein level in supernatant of co-culture of RAW264.7 cells with T cells from either control or Dex-treated mice and collected 2 days after co-culture (n = 3).

(B) Statistical results of TRAP positive RAW264.7 cell number after co-culturing with T cells of control mice, Dex-treated mice, or Dex-treated mice + anti-RANKL antibody.

(C) Murine T cell line A1.1 cells were treated with Dex at different doses or durations, then the RANKL mRNA levels were analyzed.

(D) The mRNA expression relative to beta-actin of RANKL in splenic T cells, at 2h, 4h, 8h and 20h after Dex injection.

(E) RANKL protein level in ctrl and Dex-treated A1.1 cell line detected by western blotting analysis

(F) The mRNA expression of RANKL relative to beta-actin in A1.1 cells treated with or without Dex (100 nM), in the presence or absence of inhibitor of glucocorticoid receptor RU486 (1 μM).

(G) The mRNA expression of RANKL relative to beta-actin in A1.1 with Dex (100 nM), ciclosporin A (200 ng/mL), ionomycin (100 nM). *p < 0.05; **p < 0.01; *** p <0.001; **** p <0.0001

**FIGURE S4. Proliferation and apoptosis of T cells in Dex-treated mice**

(A) Immunofluorescence staining for Ki67^+^ T cells in bone marrow of SCID and SCID - Dex after T cells were transplanted. Scale bars, 25 μM.

(B) Cell cycle distribution of splenic T cells with or without Dex treatment (n = 3).

(C) Apoptotic T cells in bone marrow and spleen, as detected by flow cytometry (n = 5).

(D) The mRNA levels of anti-apoptotic and pro-apoptotic genes relative to beta-actin in T cells were analyzed at different time points after Dex injection.
