# Supplementary figures and images for: The critical role of T cells in glucocorticoid-induced osteoporosis
Source: Cell Death Dis. 2020 Dec 14;12(1):45. doi: 10.1038/s41419-020-03249-4 (PMC7791068; doi:10.1038/s41419-020-03249-4)

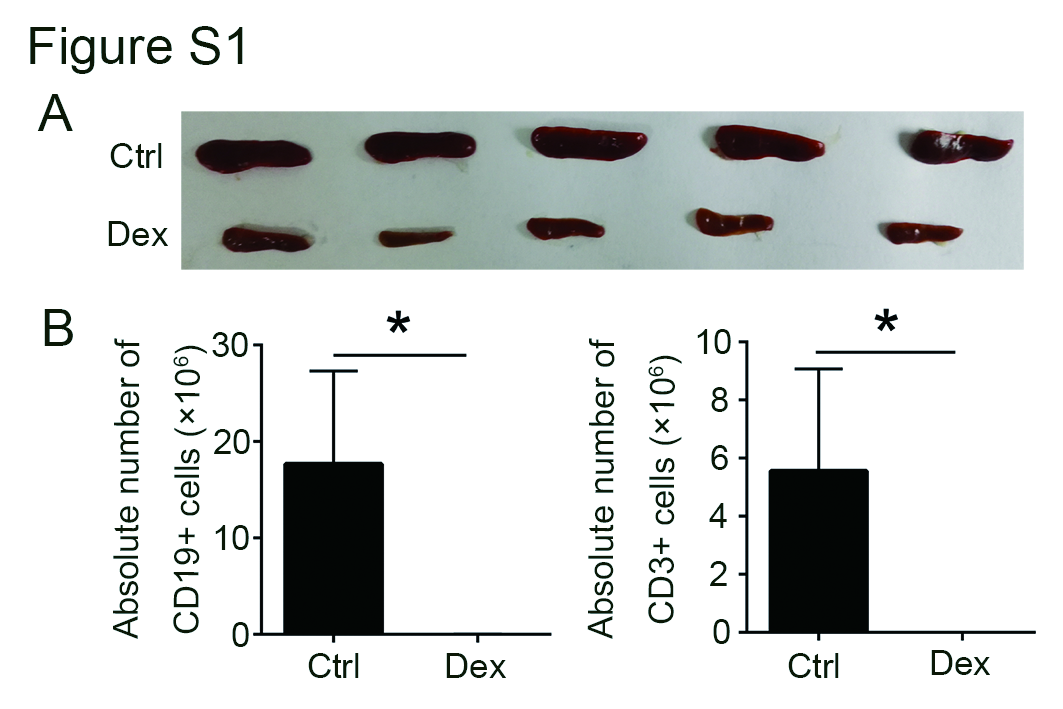

Supplement: Supplementary file 2 — FIG 1s [file 41419_2020_3249_MOESM2_ESM.tif]

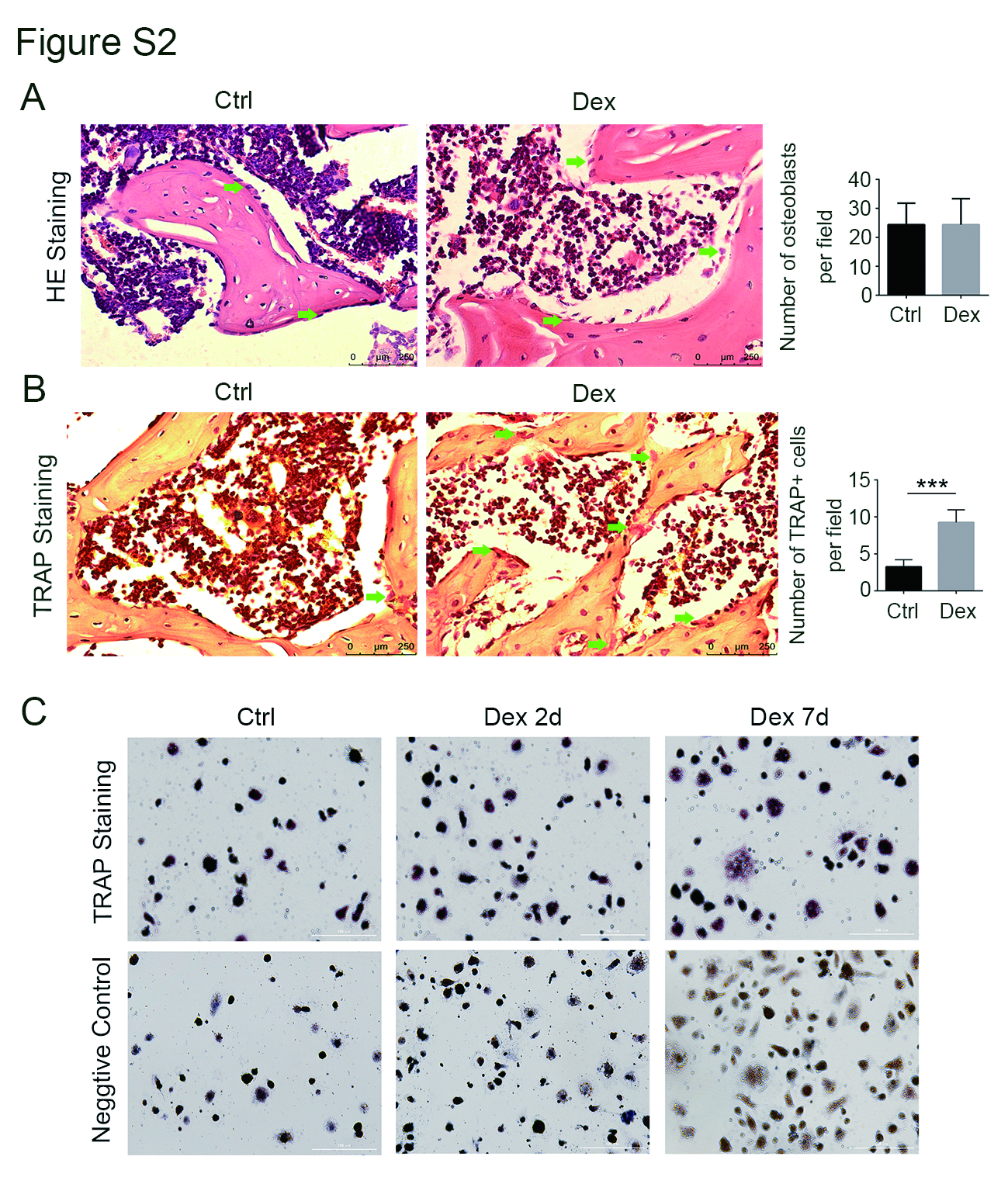

Supplement: Supplementary file 3 — FIG 2s [file 41419_2020_3249_MOESM3_ESM.tif]

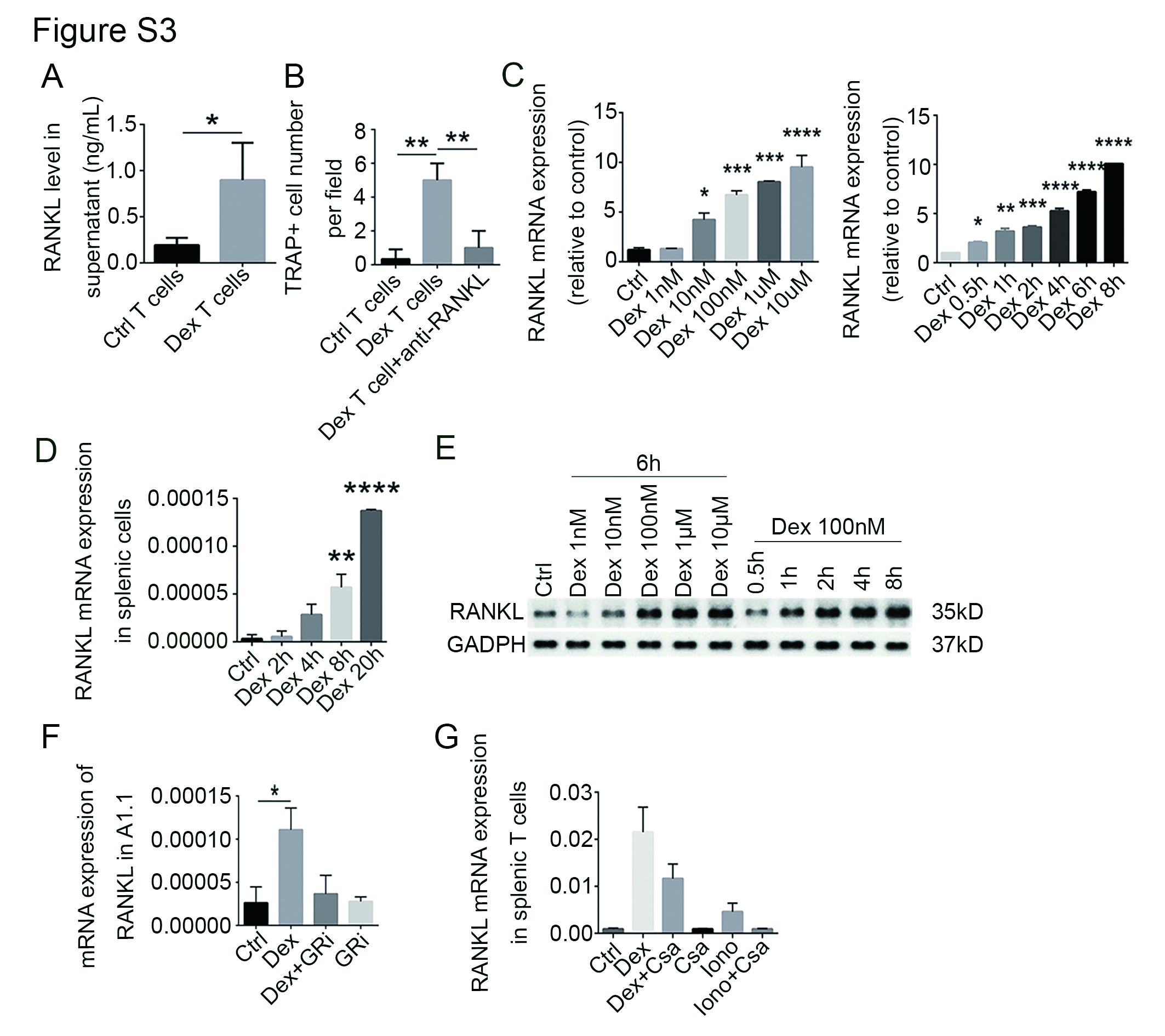

Supplement: Supplementary file 4 — FIG 3s [file 41419_2020_3249_MOESM4_ESM.tif]

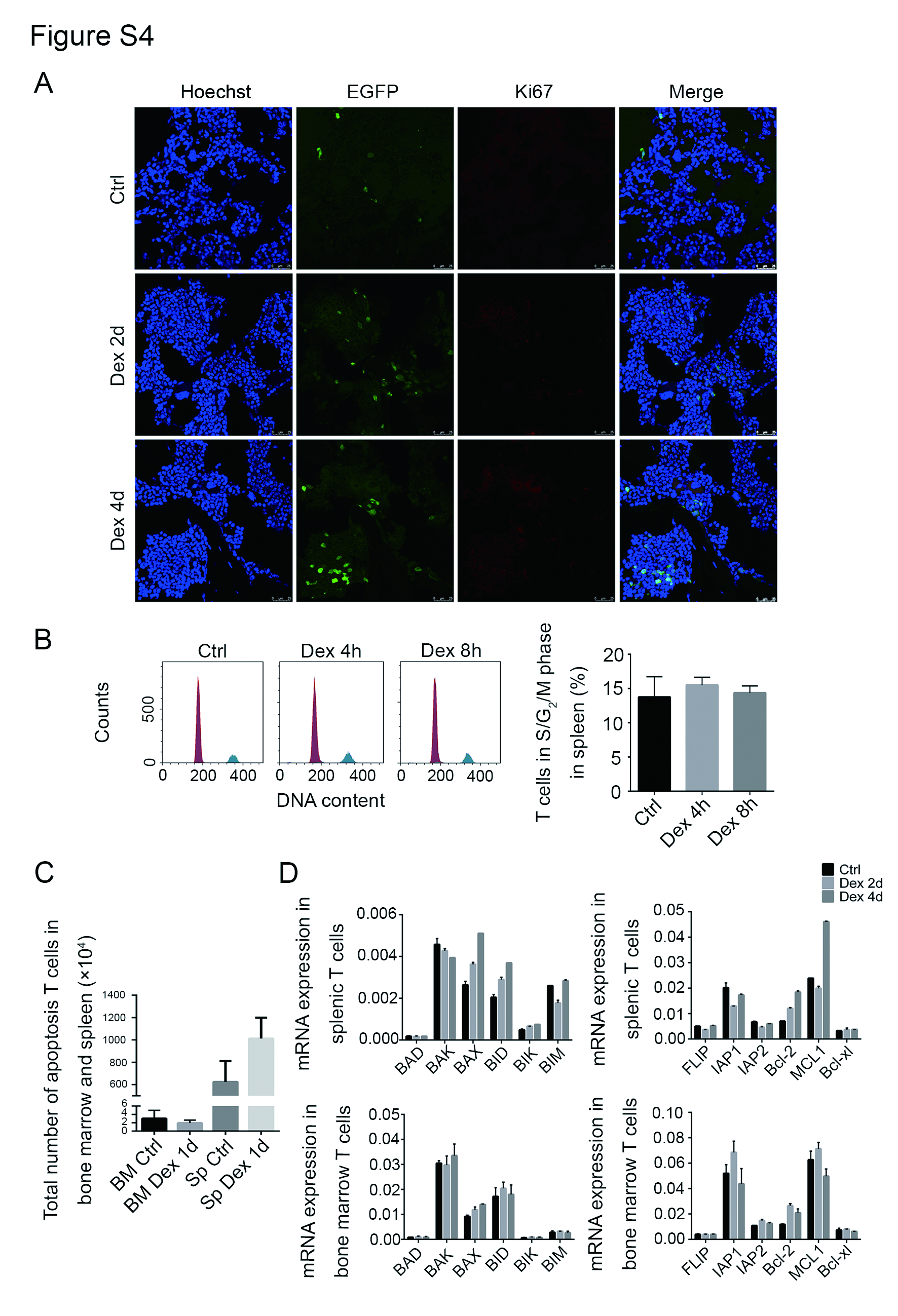

Supplement: Supplementary file 5 — FIG 4s [file 41419_2020_3249_MOESM5_ESM.tif]
